# About the Role of Interfaces on the Fatigue Crack Propagation in Laminated Metallic Composites

**DOI:** 10.3390/ma14102564

**Published:** 2021-05-14

**Authors:** Philip Manuel Pohl, Frank Kümmel, Christopher Schunk, Itziar Serrano-Munoz, Henning Markötter, Mathias Göken, Heinz Werner Höppel

**Affiliations:** 1 Materials Science & Engineering, Institute I, Friedrich-Alexander-Universität Erlangen-Nürnberg (FAU), Martensstr. 5, 91058 Erlangen, Germany; frank.kuemmel@frm2.tum.de (F.K.); christopher.schunk@fau.de (C.S.); mathias.goeken@fau.de (M.G.); hwe.hoeppel@fau.de (H.W.H.); 2 Joint Institute for New Materials and Processes (ZMP), Friedrich-Alexander-Universität Erlangen-Nürnberg (FAU), Dr.-Mack-Straße 81, 90762 Fürth, Germany; 3 Heinz Maier-Leibnitz Zentrum (MLZ), Technische Universität München (TUM), Lichtenbergstr. 1, 85748 Garching, Germany; 4 Concept Laser GmbH, An der Zeil 8, 96215 Lichtenfels, Germany; 5 Bundesanstalt für Materialforschung und-prüfung (BAM), Unter den Eichen 87, 12205 Berlin, Germany; itziar.serrano-munoz@bam.de (I.S.-M.); henning.markoetter@bam.de (H.M.)

**Keywords:** laminated metallic composites, toughening mechanisms, interfaces, fatigue crack propagation, fatigue crack growth, large chamber SEM

## Abstract

The influence of gradients in hardness and elastic properties at interfaces of dissimilar materials in laminated metallic composites (LMCs) on fatigue crack propagation is investigated experimentally for three different LMC systems: Al/Al-LMCs with dissimilar yield stress and Al/Steel-LMCs as well as Al/Ti/Steel-LMCs with dissimilar yield stress and Young’s modulus, respectively. The damage tolerant fatigue behavior in Al/Al-LMCs with an alternating layer structure is enhanced significantly compared to constituent monolithic materials. The prevalent toughening mechanisms at the interfaces are identified by microscopical methods and synchrotron X-ray computed tomography. For the soft/hard transition, crack deflection mechanisms at the vicinity of the interface are observed, whereas crack bifurcation mechanisms can be seen for the hard/soft transition. The crack propagation in Al/Steel-LMCs was studied conducting in-situ scanning electron microscope (SEM) experiments in the respective low cycle fatigue (LCF) and high cycle fatigue (HCF) regimes of the laminate. The enhanced resistance against crack propagation in the LCF regime is attributed to the prevalent stress redistribution, crack deflection, and crack bridging mechanisms. The fatigue properties of different Al/Ti/Steel-LMC systems show the potential of LMCs in terms of an appropriate selection of constituents in combination with an optimized architecture. The results are also discussed under the aspect of tailored lightweight applications subjected to cyclic loading.

## 1. Introduction

Recent studies [1,2,3,4] provide comprehensive overviews on the superior performance of heterostructured metallic materials compared to conventional metallic materials. The superior performance is based on significant synergistic effects associated with strong inter-zone interaction/coupling in the heterostructured materials [1]. The synergistic effects can be attributed to local heterogeneities and local variations of mechanical behavior in, among others, multimodal [5,6,7,8], gradient [9,10,11,12], or laminated [13,14,15,16,17] structures.

In laminated metal composites (LMCs), these local heterogeneities emerge at the interfaces between layers of different materials. Interface related mechanisms play a significant role regarding the macroscopic properties of laminated structures. For instance, the interface density, which correlates inversely with the layer thickness, was reported to have a major influence on the prevalent deformation mechanisms of laminated metal structures over several length scales of layer thickness [17,18].

Due to their layered architectures, most properties in laminated composites are inherently anisotropic. In terms of mechanical properties associated with crack growth, the most favorable properties are obtained when layers and interfaces are orientated perpendicular to the crack growth direction (crack arrester orientation) for quasistatic [19,20,21,22] as well as cyclic [23,24,25,26] loading. This can be attributed to the presence of extrinsic toughening mechanisms, resulting in a reduction of local stress intensity at the crack tip and thereby reducing the local crack driving force [27,28,29]. A comprehensive overview of different toughening mechanisms observed in laminated metal composites is provided by Lesuer et al. [27]. In crack arrester orientation, crack deflection [30,31,32], crack blunting [33], crack bridging [33], and stress redistribution mechanisms were observed in different LMC systems.

These toughening mechanisms in laminated metal composites are caused by material inhomogeneity effects [34] at the interfaces. Material inhomogeneity effects can be associated for instance with gradients in yield stress (yield stress gradient effect [35]) or elastic properties (elastic inhomogeneity effect [36,37]) at interfaces between dissimilar materials. Several numerical studies have investigated the influence of yield stress gradients on bimetal [31,35,38] as well as interlayer configurations [34,35,39]. A reduction of the local crack driving force at the vicinity of an interface has been reported when the crack approaches the interface from the softer towards the stronger layer. This was attributed to a change of the process zone size ahead of the crack upon interaction with the stronger layer and consequently leads to a crack tip shielding effect [40,41]. An anti-shielding effect was reported for the opposite case when the crack approaches the interface from the stronger to the softer layer. These findings have been confirmed experimentally [30,31,41] in bimetals subjected to constant far-field stress intensity ranges. For the soft/strong transition, the shielding effect was associated with a reduction of the fatigue crack growth rate as well as crack deflection at the vicinity of the interface. The anti-shielding effect was apparent by an increase of the fatigue crack growth rate near the strong/soft transition and a bifurcation of the crack upon entering the soft layer was observed at higher stress intensity ranges in an interlayer system [30]. Regarding the elastic inhomogeneity effect, similar shielding and anti-shielding effects have been reported at the vicinity of interfaces for compliant/stiff and stiff/compliant transitions, respectively, by numerical studies [34,42,43]. Furthermore, different numerical approaches based on cohesive zone models [44] and a strain energy density criterion [45] describe the onset of crack initiation associated with debonding at the interfaces of materials with dissimilar mechanical properties.

A recent study by Kümmel et al. [46] provides a comprehensive overview on the potential of fatigue life enhancement in specifically tailored laminated metal composites by utilizing the inherent material inhomogeneity effects at the interfaces between dissimilar materials. An increase in fatigue life in LMC systems can be accomplished by (a) enhancing resistance against crack initiation based on load transfer from surface layers into adjacent stiffer layers (gradient in elastic properties at interfaces) [15,16,46], and (b) enhancing resistance against crack propagation based on toughening mechanisms at the interfaces (gradient in hardness and elastic properties at interfaces) [15,32,47].

A variation of the stacking sequence of layers with different properties and thus the laminate architecture was reported to significantly influence fatigue life properties in laminated metal composites [15]. Fabrication of laminated metal composites can be achieved by the accumulative roll bonding (ARB) process [48], as this process allows for continuous production [49] of specifically tailored laminates [50] beyond the laboratory scale [51].

The influence of gradients in yield stress and elastic properties on (fatigue) crack growth at interfaces has been studied so far, on the one hand primarily on bimetals and interlayer systems by experimental and numerical approaches, and on the other hand on laminates, where the focus was set on the experimental determination of the fatigue lives. In this study, the influence of gradient effects at the interfaces in laminated metal composites on fatigue crack propagation and the resulting toughening mechanisms at the vicinity of interfaces is emphasized. Additionally, the lightweight application potential of LMCs with optimized architectures is addressed using different laminate architectures, which utilize the effects of gradients in yield stress and elastic properties to different extents in terms of fatigue life and resistance against crack propagation. An improved principal understanding of how these fatigue life-enhancing effects interact in LMCs is essential for an utilization in intelligently designed laminated architectures aiming for a high resistance against fatigue crack propagation.

## 2. Materials and Methods

### 2.1. Processing of the Laminated Metal Composites and Monolithic Materials

Three different systems of laminated metal composites and constituent monolithic materials for reference were produced by means of the accumulative roll bonding (ARB) process in order to study the role of (a) hardness gradients at interfaces and (b) combined gradients in hardness and elastic properties at interfaces on fatigue life and fatigue crack propagation. Regarding the first point, one laminated composite and the constituent monolithic materials were produced to additionally investigate the fatigue crack growth (FCG) properties.

All the investigated laminate and monolithic systems were roll bonded using a four high rolling mill (BW 300, Carl Wezel, Mühlacker, Germany). Prior to roll bonding, the sheet metal surfaces were cleaned with acetone and were wire brushed to remove oxide layers and to achieve sufficient bonding. Subsequently, the treated sheet metal surfaces were stacked and roll bonded at a nominal thickness reduction of 50% per ARB cycle at ambient conditions, air cooled afterwards and halved in length before performing the next cycle. The chemical composition of all sheet metal alloys used in this study is listed in Table 1.

**Figure 1:**
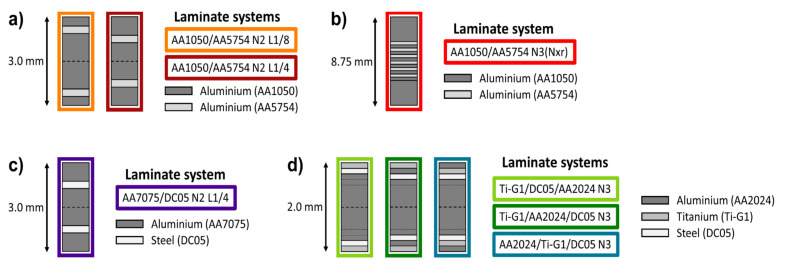
Schematic illustration of sheet materials, stacking sequence, and notation of different laminate systems. (**a**) Bi-material systems with hardness gradients at interfaces: AA1050/AA5754 N2 L1/4 and AA1050/AA5754 N2 L1/8; (**b**) bi-material system with hardness gradients at interfaces: AA1050/AA5754 N3(Nxr); (**c**) bi-material system with gradients of hardness and elastic properties at interfaces: AA7075/DC05 N2 L1/4; (**d**) tri-material systems with gradients of hardness and elastic properties at interfaces: Ti-G1/DC05/AA2024 N3, Ti-G1/AA2024/DC05 N3 and AA2024/Ti-G1/DC05.

In order to study the influence of thin layers of a stronger material embedded in a softer matrix on fatigue life and crack propagation, two different architectures of laminated composites consisting of technically pure aluminium (AA1050) and a solution hardening aluminium alloy (AA5754) as well as the constituent monolithic AA1050 and AA5754 materials were produced using two ARB cycles (N2). All sheet metals had an initial size of 300 mm in length and 100 mm in width. In the first ARB cycle, one sheet of the stronger material (AA5754) was stacked in between two sheets of the softer material (AA1050). The resulting laminate architectures after two ARB cycles with a total thickness of 3.0 mm, respectively, are shown schematically in Figure 1a. For the AA1050/AA5754 composite with a laminated architecture denoted as L1/8, the layer of the stronger material is positioned further towards the surface than for the laminate architecture denoted as L1/4. For both architectures, the volume fraction of the stronger AA5754 material layers accounted for 16.6 vol.% of the total volume of the laminates. Further details on initial stacking sequences for the first ARB cycle (N1) as well as the subsequent second cycle (N2) can be found in previous studies by the authors [15] for LMCs consisting of different constituent materials.

In order to study the influence of a periodic variation of the soft (AA1050) and strong (AA5754) layers across a laminated structure on fatigue crack growth properties, a AA1050/AA5754 laminate with a total thickness of 8.75 mm was produced. Initially, sheets of AA5754 and AA1050 were 50% cold rolled down to a thickness of 2.5 mm prior to roll bonding. In the first ARB cycle, one sheet of the stronger material (AA5754) and one sheet of the softer material (AA1050) were stacked and rolled to form bimetal sheets. For the second ARB cycle, six of these bimetal sheets were stacked and roll bonded, resulting in a laminate architecture with an alternating sequence of AA1050 and AA5754 layers and a total sheet thickness of 7.5 mm. Subsequently, in the third ARB cycle, this laminate sheet was stacked in between two 2.5 mm thick AA1050 supporting layer sheets and roll bonded. A final laminated architecture for FCG measurements with a nominal layer thickness of 312 µm was achieved. The architecture of the laminated composite is schematically shown in Figure 1b. Monolithic sheet of the constituent AA1050 and AA5754 materials were produced using two ARB cycles. In order to maintain a uniform layer structure across all roll bonding cycles for the laminated composite and better comparability of results with the monolithic materials, all sheets were fully recrystallized prior to the first, second, and if applicable third ARB cycle for 2 h at 365 °C. This ARB processing route is denoted in the following as N3(Nxr) for the laminated composite and as N2(N1r) for monolithic materials in order to account for the intermediate recrystallization steps.

The influence of a combined difference in strength and elastic properties at interfaces between layers on fatigue life and crack propagation was studied (a) on a bi-material laminate system (AA7075/DC05 laminate) and (b) on tri-material laminate systems consisting of AA2024, Ti-Grade 1, and DC05 deep drawing steel.

The ARB processing of the AA7075/DC05 N2 L1/4 architecture followed the same route as described above for the AA1050/AA5754 N2 L1/4 laminate system. The laminate architecture is depicted schematically in Figure 1c. Monolithic AA7075 N2 aluminium and DC05 N2 steel sheets were roll bonded accordingly for reference measurements. To ensure sufficient bonding, all sheets were preheated for 5 min at 280 °C prior to each roll bonding step.

For the AA2024, Ti-Grade 1 and DC05 laminate systems, three roll bonding steps were performed. For the first roll bonding step, a shell structure and a core structure were prepared for each laminate architecture. The core structure consists of four 1 mm AA2024 sheets, which were roll bonded in the first ARB cycle (N1) to form 2.0 mm thick sheets. The shell structures were stacked in three different arrangements of layers of 1.0 mm AA2024, Ti-Grade 1 and DC05 sheets. The nomenclature states the layer materials starting from the surface layer towards the inner most layer. Three different shell structures were stacked in the first ARB cycle: AA2024/Ti-G1/DC05, Ti-G1/DC05/AA2024 and Ti-G1/AA2024/DC05. All shell structures were stacked adding one additional 1.0 mm AA2024 sheet at the inner most position and subsequently roll bonded in the first ARB cycle (N1) to produce 2.0 mm thick laminate sheets. In the second ARB cycle (N2), one core structure and one shell structure were each roll bonded to form asymmetrical laminates. In the ensuing third ARB cycle (N3), two asymmetrical N2 laminate structures were roll bonded at the respective core structures to produce symmetric laminate architectures. The respective stacking sequences (architectures) of the Ti-G1/DC05/AA2024 N3, Ti-G1/AA2024/DC05 N3 and AA2024/Ti-G1/DC05 N3 laminate systems are shown in Figure 1d. Monolithic AA2024 N3, Ti-Grade 1 N3 and DC05 N3 sheets were produced accordingly.

### 2.2. Characterisation of Local Mechanical Properties

The local mechanical properties were measured by means of nanoindentation experiments (Nanoindenter XP, MTS Nano Instruments, Oak Ridge, TN, USA) with the continuous stiffness method [52] using a three-sided Berkovich pyramid. The surfaces of the samples were prepared by grinding down to a grit size of 5 µm and mechanical polishing down to 1 µm grit size using diamond suspension. This was followed up by a chemo-mechanical polishing step using SiO_2_ polishing suspension (OPS and OPU, Struers, Willich, Germany). In each sample, indentation fields of 50 to 200 indents, depending on the laminate size, were measured across the interfaces. The indentation depth was set to 1000 nm. The distance between indents was chosen to be 25-fold of the indentation depth to avoid influences of the damage zone around previous indents on the current measurement [53].

Uniaxial tensile tests were performed on a universal testing machine (Instron 4505, Instron, Darmstadt, Germany) at room temperature and a constant engineering strain rate of 10^−3^ s^−1^. The tensile specimen had a gauge section of 10 mm length (in rolling direction), 4.5 mm width (in traverse direction), and 2.0 mm or 3.0 mm height (in normal direction). For each material, three tensile tests were conducted.

### 2.3. Determination of Fatigue Life

In order to determine fatigue live properties of laminate systems and constituent monolithic materials, fatigue tests were carried out on a vibrophore testing machine (HFP 5100, Roell Amsler, Ulm, Germany) in three-point bending mode. Specimen with dimensions of 20 mm length (in rolling direction), 9.5 mm width (in traverse direction) and 2.0 mm or 3.0 mm height (in normal direction) were machined from the sheets, respectively. The surfaces were prepared by grinding down to a grit size of 5 µm before testing. Laminate samples were tested in crack arrester orientation, where interfaces are oriented perpendicular to the direction of crack propagation. The fatigue tests were conducted in force control with an R-value of 0.1 and a resulting resonance frequency of about 50 to 70 Hz, depending on the stiffness of the sample. The frequency was monitored constantly throughout testing. Experiments were terminated upon a resonance frequency drop of 5 Hz, resulting from loss of stiffness due to (macro-)crack propagation and leading to the value for the numbers of cycles to failure (N_f_). Post-experiment, the onset of crack propagation was determined using a 0.1 Hz resonance frequency drop criterion. The resulting numbers of cycles were denoted as numbers of cycles to crack initiation (N_i_).

### 2.4. Determination of Fatigue Crack Growth

**Figure 2:**
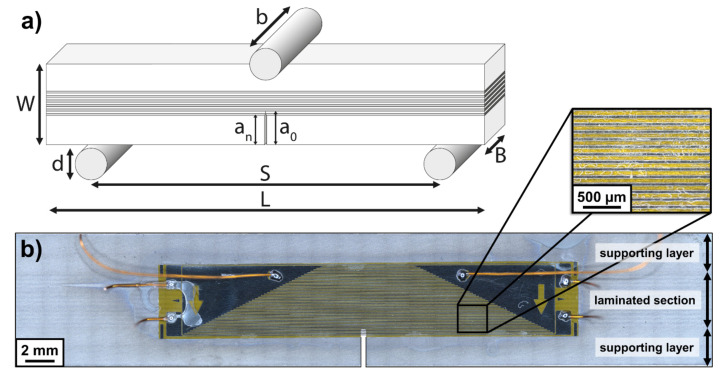
Single edge notched specimen geometry SE(B) for fatigue crack growth (FCG) measurements on laminated materials. (**a**) Notation of SE(B)-specimen dimensions; (**b**) Microscopical image of the specimen surface indicating the supporting layers and laminated structure (barely visible due to low contrast) as well as crack length measurement using crack propagation gauges.

Fatigue crack growth (FCG) measurements were conducted on a servohydraulic testing machine (MTS810, MTS System Corporation, Eden Prairie, MN, USA) using the SE(B)-specimen (single edge notched bending) geometry (see Figure 2a). In accordance with standards ASTM E1820 [54] and ISO 12135 [55], the following dimensions for the specimen and setup were satisfied in relation to the specimen width *W*: thickness *B* = 0.5 *W*, span *S* = 4 *W*, length *L* > 4.5 *W*, roller pin diameter 0.5 *W* > *d* > 0.25 *W* and roller pin length *b* > 1.25 *W*. The stress intensity factor *K* for the SE(B)-specimen geometry was calculated at a force *P* according to ASTM 1820 [52] as follows:

(1) $$K=\frac{P\;S}{B\;W^{3/2}}\;f\left(\frac{a}{W}\right),$$

with:

(2) $$f\left(\frac{a}{W}\right)=\frac{3\;{\left(\frac{a}{W}\right)}^{1/2}\left[1.99-\left(\frac{a}{W}\right)\;\left(1-\frac{a}{W}\right)\;\left(2.15-3.93\;\left(\frac{a}{W}\right)+2.7\;{\left(\frac{a}{W}\right)}^{2}\right)\right]}{2\;\left(1+2\;\frac{a}{W}\right){\left(1-\frac{a}{W}\right)}^{3/2}},$$

where the stress intensity factor *K* depends solely on the parameters describing the geometry of the complete sample as well as the crack length *a*. Thus, the crack driving force depending on the calculated stress intensity factor *K* needs to be interpreted as a far-field crack driving force for the laminated specimens, as opposed to a local driving force for concepts taking into account the local variation of the stress intensity factor in a bimetal or laminate of dissimilar materials [56,57,58].

The samples were machined from the rolled laminate and monolithic material sheets in T-S orientation (ASTM E1823 [59]) with following dimensions: *L* = 50 mm (in traverse direction), *W* = 8.75 mm (in normal direction) and *B* = 4.4 mm (in rolling direction). Subsequently, a notch with notch length *a_n_* = 0.3 *W* = 2.65 mm was introduced using wire electrical discharge machining, resulting in a notch orientated perpendicular to the interfaces (crack arrester orientation) for laminate specimen. The specimen surfaces were prepared by grinding down to a grit size of 5 µm before testing. Crack propagation gauges (FAC-5, Tokyo Measuring Instruments Lab., Tokyo, Japan) with a resolution of 0.1 mm glued to the surface of each specimen, as can be seen in Figure 2b, allowed for automated and discrete measurement of crack extension during the test across the complete laminated section of the specimen. The difference between surface crack extension and crack propagation gauge measurement was detected to be below 0.2 mm. The FCG experiments were conducted under stress intensity *K* control, an *R*-value of 0.1 and a testing frequency of 3 Hz. The *K* controlled operation was realized using a closed loop feedback of crack extension measurement to automatically adjust the force loading to achieve the respective *K* level (load shedding technique [60]). Prior to testing, the machined notch root was sharpened using razor blade grinding and subsequently pre-cracked at a constant stress intensity range of ∆*K* = 5 MPa√m to a minimum crack extension of 0.3 mm to reach the initial crack length a_0_ for the FCG tests.

The FCG tests on monolithic specimen were performed using ∆*K*-increasing and ∆*K*-decreasing procedures as well as ∆*K*-constant tests additionally. As suggested by the ASTM E647 standard [60], for ∆*K*-decreasing tests, a normalized *K*-gradient *C* > −0.08 mm^−1^ was chosen to minimize effects of prior loading history on FCG rates below 10^−5^ mm/cycle. For ∆*K*-increasing tests, the normalized *K*-gradient was set below *C* < 0.08 mm^−1^ to minimize the influence of additional transient crack growth effects. ∆*K*-constant tests were only performed on laminate specimen, as the external driving force for crack growth remains constant and changes in process zone size ahead of the crack tip only depend on local conditions associated with the laminated structure of materials with dissimilar mechanical properties. This aids in terms of a better understanding of effects on FCG properties associated with toughening mechanisms arising from interactions between the process zone ahead of the crack tip and the laminated structure. The FCG experiments were terminated as the cracks approached the end of the laminated structure. The crack growth rate *da/dN* was calculated as the average crack growth rate throughout the laminated section 0.3 < *a/W* < 0.7 of each specimen (Figure 2) tested at a certain ∆*K* level.

### 2.5. Characterisation of Damage Mechanisms

In order to identify damage mechanisms in laminated structures post-mortem, the surface crack networks of the fatigued specimen were examined by scanning electron microscopy (Crossbeam 1540 EsB, Zeiss, Oberkochen, Germany) using secondary and backscattered electron contrast techniques as well as light microscopy (Axio Imager M1, Zeiss, Oberkochen, Germany) using brightfield and dark field contrast techniques.

Synchrotron X-ray computed microtomography (SXCT) experiments were performed at the BAMline [61] at the electron storage ring Bessy II (HZB, Berlin, Germany) on the crack network of a AA1050/AA5754 N3(Nxr) laminate specimen after the FCG experiment at constant ∆*K* = 17.5 MPa√m. A cuboidal sample with a cross section of 4 × 4 mm^2^ (width *W* × thickness *B*) and a length L of 20 mm containing the entire crack network was extracted metallographically from the laminated section of the SE(B)-specimen. Two scans were performed along the sample: One corresponding to the path of the primary crack network and another one adjacent to the prior scan to further enlarge the field of view to display the secondary crack network. The energy of the monochromatic and parallel beam was set to 30 keV using a double multilayer monochromator (DMM) with an energy resolution of ~3%. An effective pixel size of 2.2 µm was chosen using a corresponding microscope objective (2× magnification, Olympus, Hamburg, Germany) in an X-ray microscope setup (Optique-Peter, Lentilly, France) combined with a charge-coupled device (CCD)-based camera (4008 × 2672 pixels, PCO, Kelheim, Germany). Each tomographic scan comprised 2416 projections recorded within a total scan time of 2.5 h. The distance between the scintillator screen of the detector (~60 µm CdWO_4_) and the investigated samples was 10 mm. An in-house routine developed on Python software [62] was used to reconstruct the projections. The reconstructed volume data was denoised using a non-local means filter, with Fiji software [63]. Subsequently, the cracks were identified by global threshold segmentation. Visualization of the segmented cracks was performed using the AvizoFire 9.4 software package [64].

### 2.6. In-Situ Characterisation of Damage Mechanisms

In order to gain additional information on crack propagation at the vicinity of interfaces and to investigate the prevalent damage mechanisms, specimen of the AA7075/DC05 N2 L1/4 laminate system were fatigued in-situ in a large chamber scanning electron microscope (LC-SEM, Visitec, Grevesmühlen, Germany), see [65,66,67] for details. Therefore, specimen with dimensions of 30 mm length (in rolling direction), 9.5 mm width (in traverse direction) and 3.0 mm height (in normal direction) were machined from the AA7075/DC05 N2 L1/4 laminate sheet. Prior to testing, the surfaces of the samples were prepared by grinding down to a grit size of 5 µm and mechanical polishing down to 1 µm grit size using diamond suspension. In-situ fatigue experiments in three-point bending mode were conducted in the LCF regime at a maximum stress amplitude ∆*σ_max_*/2 = 295 MPa as well as in the HCF regime at ∆*σ_max_*/2 = 210 MPa. In order to study crack propagation in the HCF regime in-situ, the specimen was pre-fatigued on a vibrophore testing machine (HFP 5100, Roell Amsler, Ulm, Germany) at ∆*σ_max_*/2 = 210 MPa. The pre-cycling test was terminated after 9.1 Mio cycles using a 0.1 Hz resonance frequency drop criterion, indicating the end of the crack nucleation phase.

**Figure 3:**
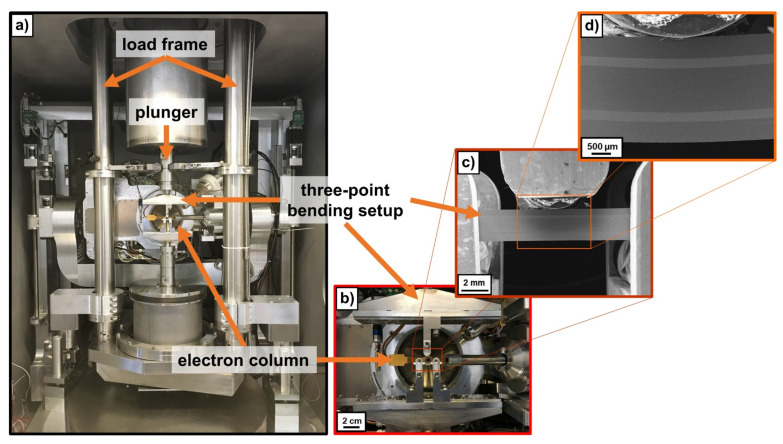
In-situ fatigue testing setup inside the large chamber scanning electron microscope (LC-SEM): (**a**) Electron gun oriented perpendicular to the front surface of specimen positioned inside the servohydraulic MTS810 testing machine; (**b**–**d**) Three-point bending setup and AA7075/DC05 N2 L1/4 laminated composite specimen at different magnifications, respectively.

The in-situ fatigue tests were conducted on a servohydraulic testing machine (MTS810, MTS System Corporation, Eden Prairie, MN, USA) which can be installed inside the vacuum chamber of the LC-SEM (Figure 3a). The electron gun was tilted at 90° and thus oriented perpendicular to the front surface of the specimen. This allowed for in-situ monitoring of crack nucleation and crack propagation at the front surface of the specimen during fatigue experiments (Figure 3b–d).

The as prepared laminate sample for LCF fatigue testing and pre-fatigued laminate sample for HCF fatigue testing were positioned on the three-point bending fixture accordingly and the vacuum chamber was evacuated to a pressure below 5 × 10^−6^ mbar for SEM operation. The fatigue tests were conducted in force control with an *R*-value of 0.1 and a testing frequency of 1 Hz. The crack propagation was monitored in-situ by scanning electron microscopy. At certain intervals, the fatigue test was interrupted to characterize the current state of crack propagation (interrupted monitoring) using the secondary electron contrast technique before continuing the fatigue experiment. Therefore, a constant loading of 0.2 *σ_max_* was applied on the one hand to open the crack wakes for SEM characterization as well as on the other hand to minimize possible transient crack growth effects during these periods of interrupted monitoring.

## 3. Results

### 3.1. Effects of a Hardness Gradient at Interfaces on Fatigue Life and Crack Propagation in LMCs

The AA1050/AA5754 N2 LMC systems were investigated in order to study the effects of integrating thin layers of a stronger material embedded in a softer matrix on the fatigue life and crack propagation. The local mechanical properties of the different layer materials after N2 processing can be seen in Table 2.

The hardness was calculated as a mean value of the individual AA1050 and AA5754 layers, respectively, from both the L1/4 and L1/8 architecture. The hardness of the AA1050 layers is about 0.8 GPa and of the AA5754 layers about 1.4 GPa. Consequently, the hardness gradient at the laminate interfaces can be calculated to be about 0.6 GPa. However, it must be noted, that this estimation of hardness gradient at laminate interfaces does not take into account the formation of an interface affected zone at the immediate vicinity of an interface caused by different shearing behavior of dissimilar materials [14,68,69]. This must also be considered for the nanoindentation results presented in the following sections.

**Figure 4:**
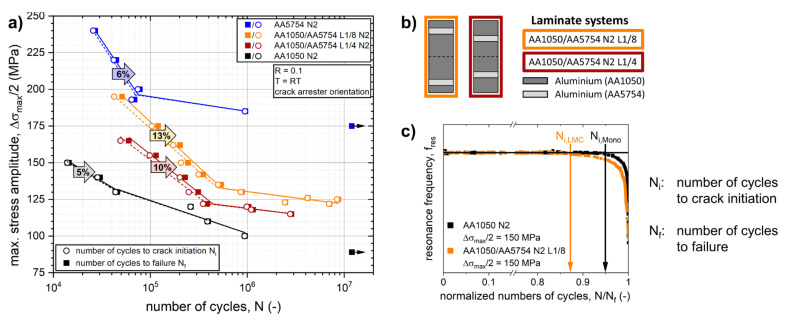
(**a**) Fatigue properties (*S-N* curves) of different AA1050/AA5754 N2 LMC architectures and constituent monolithic materials. Data partly adapted from [15,32,46]. The percentile of fatigue crack propagation on total fatigue life is indicated in the respective LCF regimes; (**b**) schematic illustration of the LMC architectures; (**c**) determination of the onset of fatigue crack propagation in the LCF regime for a monolithic and LMC sample using a 0.1 Hz resonance frequency drop criterion.

The fatigue life diagrams (*S-N* curves) of AA1050/AA5754 N2 laminate systems with two different architectures as well as of the constituent monolithic AA1050 N2 and AA5754 N2 materials are plotted in Figure 4a. For each specimen tested, the number of cycles to crack initiation (*N_i_*) as well as the numbers of cycles to failure (*N_f_*) are shown. The determination of the number of cycles to crack initiation using a 0.1 Hz drop criterion of resonance testing frequency can be seen in Figure 4c for a monolithic AA1050 sample and a AA1050/AA5754 laminated composite.

As can be seen in Figure 4a, the fatigue life of a soft AA1050 matrix material can be enhanced significantly by integrating thin and strong interlayers of AA5754 forming a laminated structure. This can be observed in both the LCF and the HCF regimes. The fatigue life of the L1/4 architecture is lower for both regimes compared to the L1/8 architecture, where the harder AA5754 layer is positioned more towards the surface (see Figure 4b). The increased fatigue life for the L1/8 architecture might be caused by effects of the interface affected zone [14,68,69], different shear strain distribution in the respective laminate surface layers during ARB processing [70] or a complex internal stress state upon cyclic loading due to the co-deformation of different aluminium layers with dissimilar hardness [71]. The transition between the LCF and HCF regime is correlated to a threshold value which depends on the material of the layer at the surface [16,32,46]. Below the threshold, in the HCF regime, the fatigue life of monolithic materials and laminated composites is primarily determined by the fatigue crack initiation stage: The numbers of cycles to crack initiation account for 95% to 99% of the numbers of cycles to failure for both monolithic materials and laminates. Above the threshold, in the LCF regime, the role of the crack propagation stage of fatigue life is significantly enhanced for the laminated composites compared to monolithic materials. The crack propagation stage accounts for 10% of numbers of cycles to failure for the AA1050/AA5754 N2 L1/4 composite and 13% for the AA1050/AA5754 N2 L1/8 composite compared to about 5 to 6% for the monolithic AA1050 N2 and AA5754 N2 materials, respectively.

Figure 4c shows a comparison of the drop of the resonance testing frequency between a laminated AA1050/AA5754 N2 L/8 specimen and a monolithic AA1050 N2 specimen tested in the LCF regime at 150 MPa (i.e., around 20 MPa above the threshold value). In both samples, the initiation of the macro-crack occurs at the respective surface layers consisting of the softer AA1050 material. As visible in the diagram, the resonance frequency of the laminated structure initially drops significantly slower with respect to the normalized numbers of cycles to failure *N/N_f_* as compared to the monolithic sample. This behavior can be attributed to a retardation of crack propagation at the vicinity of the interface to the harder AA5754 layer in the laminated composite [46]. Moreover, macro-crack propagation captures a significantly higher fraction of the total fatigue life in the LMCs than for the monolithic materials, as indicated by *N_i,LMC_* and *N_i,Mono_* in Figure 4c.

### 3.2. Effects of a Hardness Gradient at Interfaces on Fatigue Crack Growth (FCG) in LMCs

The effects of a periodic variation of strong and soft layers in a laminated composite and the associated result of having a periodic variation of the local driving forces at crack tips near the interfaces were studied using fatigue crack growth experiments on a AA1050/AA5754 N3(Nxr) laminate architecture. The local hardness of the different layers in the laminated section of the LMC architecture are listed in Table 3 as mean values of all AA1050 and AA5754 layers, respectively.

As shown in Table 3, no significant difference in hardness between AA1050 and AA5754 layers in the N3(Nxr) processed laminate and the hardness in the respective N2(N1r) processed monolithic materials can be observed. As the laminate and monolithic materials were recrystallized prior to each roll bonding process, this indicates that the local mechanical properties of the respective laminate layers and their constituent monolithic material can be considered comparable. The AA5754 layers in the LMC exhibit a hardness of 1.35 GPa and will be denoted as the stronger/harder layers in the following in opposition to the softer AA1050 layers with a hardness of 0.73 GPa. The resulting hardness gradient at the interfaces in the laminated composite can be calculated to be about 0.6 GPa.

**Figure 5:**
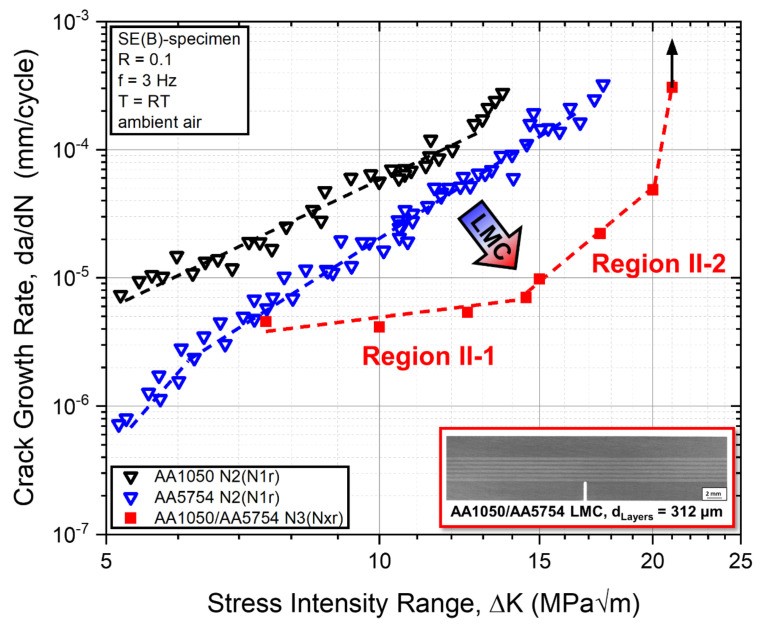
Fatigue crack growth rates in a AA1050/AA5754 N3(Nxr) laminated composite (crack arrester orientation) with a hardness gradient at interfaces and constituent monolithic materials AA1050 N2(N1r) and AA5754 N2(N1r) as a function of the applied stress intensity range ∆*K*.

Fatigue crack growth rates from increasing, decreasing, and constant stress intensity range tests for monolithic AA1050 and AA5754 as well as for constant stress intensity range tests for the AA1050/AA5754 laminated composite in crack arrester orientation are shown in Figure 5.

No significant differences between crack growth rates measured by increasing, decreasing, and constant stress intensity range tests were found for monolithic AA1050 and AA5754 specimen, respectively. This suggests that there are no effects on the crack growth rate from prior loading history for decreasing tests and from transient crack growth effects for increasing tests, since the normalized *K*-gradients for each test were chosen appropriately. The crack growth rate, da/dN, in monolithic materials between 5 and 12.5 MPa√m for AA1050 and 6–16 MPa√m for AA5754 can be described with respect to the applied stress intensity range, ∆*K*, by the well-known Paris power-law relationship [72,73]:

(3) $$\frac{da}{dN}=C\;\Delta K^{m},$$

where *C* and *m* are the scaling constants, characterizing the crack growth behavior in Region II. The Paris equation exponents *m* were found to be about *m* = 3.4 and 4.5 for the monolithic AA1050 and AA5754 materials, respectively (see Table 4). The crack growth rates of the monolithic AA5754 material are lower compared to the AA1050 alloy. At a stress intensity range below about 6 MPa√m, a deviation from Region II crack growth behavior can be observed for AA5754, indicating the transition towards near-threshold Region I fatigue crack growth. This suggests that the threshold ∆*K_th_* of the technically pure aluminium AA1050 is lower than that of the AA5754 alloy. The near threshold fatigue crack growth behavior of the materials was not addressed experimentally, as measurement of crack growth rates below 5 × 10^−7^ mm/cycle were associated with long measurement times at a testing frequency of 3 Hz on the servohydraulic testing system. Additionally, measurements of crack growth rates above 3 × 10^−4^ mm/cycle were associated with increasing effects of plasticity resulting in mixed mode failure of specimen from plastic collapse and fatigue crack growth. The measurement data above this threshold value was discarded, as the crack length measured by the crack propagation gauges was considered inaccurate.

Regarding the laminated composite, the crack growth behavior between 7.5 and 20 MPa√m can be divided into two different zones, which are denoted as Region II-1 and Region II-2 in the following. The Paris equation exponents *m* of the laminated composite were calculated to be to be about *m* = 0.9 and 5.7 for Region II-1 and Region II-2, respectively (Table 4).

As shown in Figure 5, the fatigue crack growth rate of the laminated AA1050/AA5754 composite is significantly reduced compared to crack growth rates in both constituent monolithic materials. In Region II-1, the fatigue crack growth rate in the laminated composite at a constant ∆*K* of 7.5 MPa√m is about the same as in the monolithic AA5754 and about 20% of the crack growth rate in monolithic AA1050 material. For FCG tests at higher constant ∆*K* levels in Region II-1, further deviation of crack growth rates between the LMC and monolithic constituent material can be observed, as indicated by the lower exponent *m* for the laminated composite. At ∆*K* = 14.5 MPa√m, the fatigue crack growth rate behavior changes and can be described at subsequent higher stress intensity ranges using a different exponent *m* for Region II-2. FCG tests at the transition between Regions II-1 and II-2 indicate the biggest decrease of fatigue crack growth rate in the laminated composite compared to the constituent materials (Figure 5). The FCG rate of the LMC at 14.5 MPa√m was measured to be about 7% of the crack growth rate in monolithic AA5754 material.

A reduction of the fatigue crack growth rate in a laminated composite tested in crack arrester orientation of this magnitude compared to the constituent monolithic materials can only be explained by crack growth retardation effects associated with toughening mechanisms. In LMCs, these toughening mechanisms emerge from interactions between the process zone ahead of the crack tip and the periodic variation of mechanical properties at the interfaces due to the laminated architecture [27].

**Figure 6:**
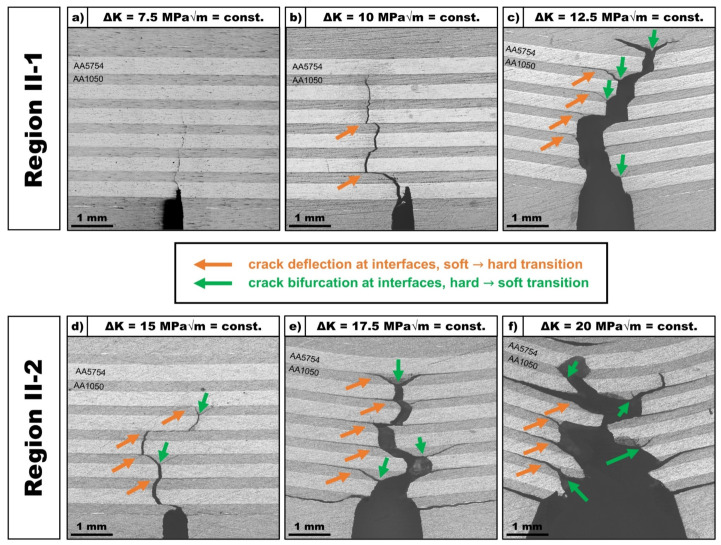
Light microscopical images of the surface crack networks in AA1050/AA5754 N3(Nxr) laminated composite structures consisting of soft AA1050 and hard/strong AA5754 layers. Identification of different toughening mechanisms at LMC interfaces resulting in impeded fatigue crack growth in crack arrester orientation for (**a**–**c**) Region II-1 FCG at constant ∆*K* = 7.5 MPa√m, 10 MPa√m and 12.5 MPa√m, respectively; (**d**–**f**) Region II-2 FCG at constant ∆*K* = 15 MPa√m, 17.5 MPa√m and 20 MPa√m, respectively.

The surface crack networks of the LMC specimen were investigated after testing in order to assess the crack growth behavior perpendicular to the interfaces of the laminated composites. Figure 6 shows the fatigue crack growth paths in the laminated composite structure for experiments conducted at constant stress intensity ranges in Region II-1 (7.5 MPa√m to 12.5 MPa√m) and Region II-2 (15 MPa√m to 20 MPa√m), respectively.

At a low stress intensity range of 7.5 MPa√m, the crack path is orientated perpendicular to the layers and interfaces without being deflected significantly throughout the laminated structure. For increasing stress intensity ranges starting at 10 MPa√m, the crack path across the laminated section is increasingly impeded at the vicinity of interfaces. For experiments at constant stress intensity ranges of 12.5 MPa√m and above, a clear distinction of different toughening mechanisms at the interfaces can be observed: (a) crack deflection (orange arrows, Figure 6) can be observed when the crack approaches interfaces from the softer (AA1050) layers towards the harder (AA5754) layers and (b) crack bifurcation (green arrows, Figure 6) can be found when the crack approaches the interfaces from the harder towards the softer layers.

The crack deflection mechanism at the interface results in crack growth along the interface. As the experiments were conducted using the SE(B)-specimen geometry, the maximum bending stress is located at the symmetry plane above the notch root and is reduced along the span *S*. This limits the crack growth along the interfaces, as the driving force is reduced gradually. The deflection of the crack path at the interfaces, when cracks approach interfaces from the soft layers towards the harder layers, and consequent crack growth along the interface imply, that further crack extension along the loading axis requires a new crack to be nucleated in the adjacent layer. As this crack re-nucleation phase depends on the local (micro-) structural characteristics and the tests were operated under a constant far-field stress intensity range, it is evident that the toughening mechanism of crack deflection strongly promotes the damage tolerant fatigue behavior of the LMC.

Bifurcation of the cracks can be observed for the opposite case, where a crack approaches the interface from the harder towards the softer layer. This mechanism leads to a branching of the original crack front into two new separate cracks. The bifurcation angles are found to be around 45° in relation to the symmetry plane above the notch root, where the bending stress is at a maximum. Bifurcation of the crack front leads to local redistribution of the far-field crack driving force that is remotely applied using a constant stress intensity range ∆*K*, as it is reallocated across multiple crack tips. Crack growth across multiple crack fronts reduces the overall rate of fatigue crack growth and thus further enhances the damage tolerant fatigue properties of the laminated composite as seen in Figure 5.

**Figure 7:**
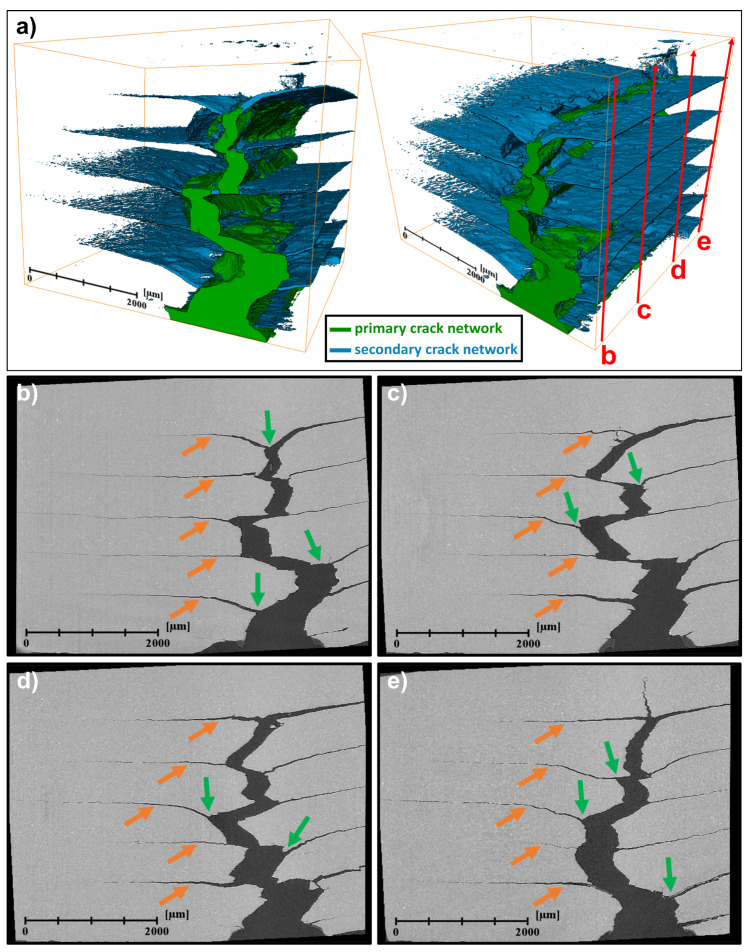
Synchrotron X-ray computed microtomography (SXCT) on a AA1050/AA5754 N3(Nxr) laminated composite specimen fatigued at a constant stress intensity range of ∆*K* = 17.5 MPa√m. (**a**) 3D-reconstruction of the crack network of the LMC specimen indicating the separation into a primary and secondary crack network; (**b**–**e**) cross sections extracted from the 3D-tomogram at different positions *z* = 0.05 *B*, *z* = 0.33 *B*, *z* = 0.66 *B* and *z* = 0.95 *B* (indicated by red arrows in (**a**)) in relation to the specimen thickness *B*, respectively. Identification of the toughening mechanisms (**a**) crack deflection (orange arrows) and (**b**) crack bifurcation (green arrows) throughout the crack networks in all cross sections.

Further investigation into these toughening mechanisms was done using synchrotron X-ray computed microtomography (SXCT), as these mechanisms described above were identified analyzing the crack networks at the surfaces of the laminated composites post-mortem. Results of the SXCT experiment on the AA1050/AA5754 N3(Nxr) laminated composite specimen fatigued at a constant stress intensity range of ∆*K* = 17.5 MPa√m, as can be seen in Figure 7.

The 3D-reconstruction of the crack network in the laminated specimen (Figure 7a) shows that the crack network can be divided into a primary (green) and a secondary (blue) crack network. The primary crack network starts at the notch root and subsequently grows along the symmetry plane of the laminated SE(B)-specimen. At this symmetry plane, the bending stress is at a maximum and promotes the highest tensile stresses perpendicular to the direction of the crack path and thus the highest driving forces at the crack tip. At the vicinity of the interfaces, the crack path is deflected due to interaction mechanisms between the process zone ahead of the crack tip and the variation of local mechanical properties at the interfaces. This results in the formation of the secondary crack network associated with the prevalent toughening mechanisms of crack deflection and crack bifurcation.

The crack networks in four cross sections of the 3D-tomogram at different positions *z* = 0.05 *B*, *z* = 0.33 *B*, *z* = 0.66 *B* and *z* = 0.95 *B* across the thickness *B* of the LMC specimen were analyzed in Figure 7b–d, respectively. Crack deflection (orange arrows) and crack bifurcation mechanisms (green arrows) can be conclusively identified in cross sections of the crack network both near the surfaces (Figure 7b,e) as well as in the volume (Figure 7c,d) of the laminated composite sample.

These findings emphasize the magnitude of the effects on damage tolerant fatigue crack growth behavior in LMCs that toughening mechanisms at interfaces in laminated metallic composites with dissimilar hardness can produce.

### 3.3. Effects of a Combined Gradient of Hardness and Elastic Properties at Interfaces on Fatigue Life and Crack Propagation in LMCs

Investigations into the influence of a combined difference in strength and elastic properties at interfaces between layers on fatigue life and the role of toughening mechanisms at interfaces on crack propagation was studied on a AA7075/DC05 N2 L1/4 laminated composite architecture. The local mechanical properties in terms of hardness and Young’s modulus of the different layer materials after N2 processing as well as the resulting gradients at interfaces can be seen in Table 5.

The average hardness was measured to be 1.55 GPa in the AA7075 aluminium layers and 2.66 GPa in the DC05 steel layers of the laminated composite. Consequently, the hardness gradient ∆*H* at the laminate interfaces can be calculated to be about 1.1 GPa. The Young’s moduli of the respective layers were assumed to be 70 GPa for AA7075 and 210 GPa for DC05 based on literature data [74,75], resulting in a gradient in elastic modulus ∆*E* of 140 GPa at the AA7075/DC05 interfaces.

**Figure 8:**
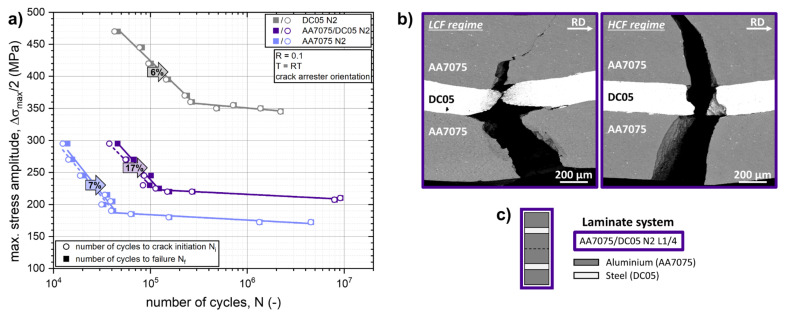
(**a**) Fatigue properties (*S-N* curves) of the AA7075/DC05 N2 L1/4 LMC architecture and constituent monolithic materials. The percentile of fatigue crack propagation on total fatigue life is indicated in the respective LCF regimes; (**b**) Fatigue crack propagation paths in AA7075/DC05 LMCs fatigued in the LCF and HCF regime, respectively; (**c**) Schematic illustration of the LMC architecture.

Figure 8a plots the fatigue life diagrams (*S-N* curves) of the AA7075/DC05 N2 L1/4 laminate architecture as well as of the constituent monolithic AA7075 N2 and DC05 N2 materials. Again, the number of cycles to crack initiation (*N_i_*) as well as the numbers of cycles to failure (*N_f_*) are shown for each fatigue test. The percentile of the fatigue crack propagation phase on the total fatigue life is specified for the LCF regimes of the laminate and monolithic materials, respectively. The S-N diagram reveals that the fatigue life of the AA7075 N2 matrix material can be enhanced in the LCF regime as well as in the HCF regime by integration of a thin DC05 steel layer.

Previous studies by the authors [15,16] on different laminated Al/Steel composite systems with the same architecture as in Figure 8c revealed that the enhancement of the endurable maximum stress amplitudes ∆*σ_max_*/2 in the HCF regime correlates with the reduction of the maximum bending stress in the outer aluminium layer due to load transfer associated with the higher elastic modulus of the steel layer. FEM analysis of the stress distribution upon loading bending of Al/Steel laminated beams showed that the introduction of the steel layer near the surface of the laminate reduces the tensile stress at the aluminium surface layer. Using the FEM model presented by Kümmel et al. [15], the tensile stress at the outer AA7075 layer can be calculated to be reduced by 19% for the L1/4 architecture (Figure 8c) compared to monolithic AA7075 material. The fatigue limits of the AA7075/DC05 N2 L1/4 composite and the AA7075 N2 monolithic material can be estimated at 210 MPa and 170 MPa, respectively. Calculating an effective fatigue limit of the AA7075/DC05 composite and taking into account the FEM-calculated 19% reduction of the maximum tensile stress in the outer AA7075 layer resulting from the load transfer into the adjacent DC05 steel layer, the resulting effective endurable maximum stress amplitude ∆*σ_max,corr._*/2 amounts to 170.1 MPa. This fits perfectly to the obtained fatigue limit of the monolithic AA7075 N2 material of 170 MPa. The numbers of cycles, which are consumed to initiate a crack in the HCF regime, amount to 96–99% of *N_f_* for both the laminated composite and constituent monolithic materials. Again, it becomes evident that in the HCF regime the fatigue life of the LMC is primarily determined by the fatigue crack initiation phase, as is the case for the monolithic AA7075 and DC05 materials and the overall role of crack propagation processes in the LMCs subjected to loadings in the HCF regime, which is relatively small.

This load transfer effect enhances the fatigue life of the LMC in the LCF regime as well, compared to the monolithic AA7075 material. Additionally, as reported in Section 3.1. for the Al/Al LMC systems, in the LCF regime, the role of fatigue crack propagation is promoted in the AA7075/DC05 LMC system compared to the constituent monolithic materials. As indicated in Figure 8a, the crack propagation phase accounts for 17% of the numbers of cycle to failure for the AA7075/DC05 composite compared to about 5 to 7% for the monolithic AA7075 N2 and DC05 N2 materials. This increased percentile of the crack propagation phase on the overall fatigue life in the laminated composite must be associated with toughening mechanisms impeding the propagating crack front at the vicinity of the interfaces.

Figure 8b shows SEM images of the fatigue crack propagation paths through the laminated structure of the AA7075/DC05 N2 L1/4 architecture of specimens fatigued in the LCF and HCF regime, respectively. A clear difference in the crack propagation behavior in the LMC architecture between the LCF regime compared to the HCF regime can be seen. In the HCF regime, the crack path propagates relatively straight through both the interfaces. A slight deviation in crack path trajectory away from the initial orientation perpendicular to the interfaces can be observed when the crack approaches the interface towards the harder and stiffer steel layer. Before penetrating the steel layer, the crack propagates about 100 µm along the first interface. In the LCF regime, a more distinct crack network can be observed. Crack propagation in 45° orientation towards the surface can be observed in the outer AA7075 layer. Some degree of necking occurring in the DC05 steel layer hints to promoted activities associated with plasticity involved in the crack propagation process at this stage. Applying only a post-mortem analysis of the crack networks in the laminated AA7075/DC05 composites does not deliver sufficient evidence to identify individual toughening mechanisms associated with the enhanced role of fatigue crack propagation in the LCF regime.

In order to gather a better understanding of the prevalent toughening mechanisms obstructing fatigue crack propagation at the vicinity of interfaces in AA7075/DC05 N2 L1/4 laminated composites, in-situ LCF and HCF fatigue experiments have been conducted inside the large chamber SEM.

**Figure 9:**
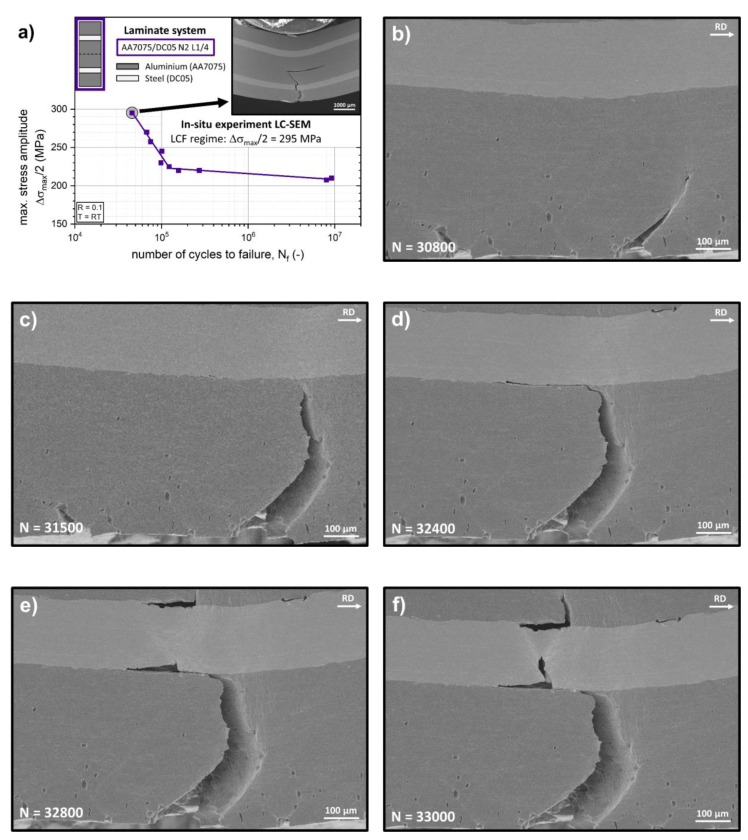
(**a**) Three-point bending fatigue experiment on a AA7075/DC05 laminated composite specimen fatigued in the LCF regime at a maximum stress amplitude of 295 MPa inside a large-chamber SEM; (**b**–**f**) In-situ observation of the fatigue crack propagation perpendicular to the interfaces (crack arrester orientation).

The experiment in the LCF regime was conducted at a maximum bending stress amplitude ∆*σ_max_*/2 of 295 MPa as shown in Figure 9a. Figure 9b shows the crack network after 30.8k loading cycles. Multiple small cracks can be observed at the front surface. The formation of these small cracks in a AA7075 alloy is known to be associated with the brittle behavior of small intermetallic particles (e.g., Mg_2_Si and Al_7_Cu_2_Fe) [76]. Two larger cracks have been formed at the bottom surface of the specimen at this stage. The larger crack on the right side was nucleated at the surface aluminium layer below the loading anvil. At this position, the tensile stress at the surface is at a maximum, resulting from the cyclic loading in three-point bending mode. This crack has propagated through half of the outer AA7075 layer at an angle of about 45° with respect to the orientation of the interface. From this position on, the crack path trajectory changes and the crack propagates perpendicular to the interfaces (Figure 9c). As the crack approaches the immediate vicinity of the interface towards the steel layer after 31.5k loading cycles, the crack path gets deflected prior to the crack tip reaching the interface (Figure 9c). Upon reaching the aluminium/steel interface, crack propagation along the interface of about 350 µm was observed during the subsequent sequence of approximately 1k loading cycles (Figure 9d).

During this period, the formation of interface delamination cracks associated with stress redistribution effects at the opposing steel/aluminium interface was observed. After 32.8k loading cycles, further crack propagation into the inner AA7075 layer of the LMC architecture starting at one of the delamination cracks was observed (Figure 9e) before the original crack begins to penetrate the steel layer. This crack bridging mechanisms of the steel layer leads to the formation of a complex crack network with simultaneous crack propagation occurring in the aluminium and steel layers (Figure 9f, 33k loading cycles). The crack propagation in the DC05 steel layer is associated with visible plastic activity around the crack tip. Although no necking of the steel layer in the in-situ LCF experiment was found, as observed in Figure 8b, the plasticity is indicated by the formation of shear bands ahead of the crack tip at an angle of about 45° to 60° in relation to the direction of loading in Figure 9f. Upon coalition of the two individual cracks, the test was stopped, as the crack network reached the bonding plane of the final N2 ARB processing step (Figure 9a).

**Figure 10:**
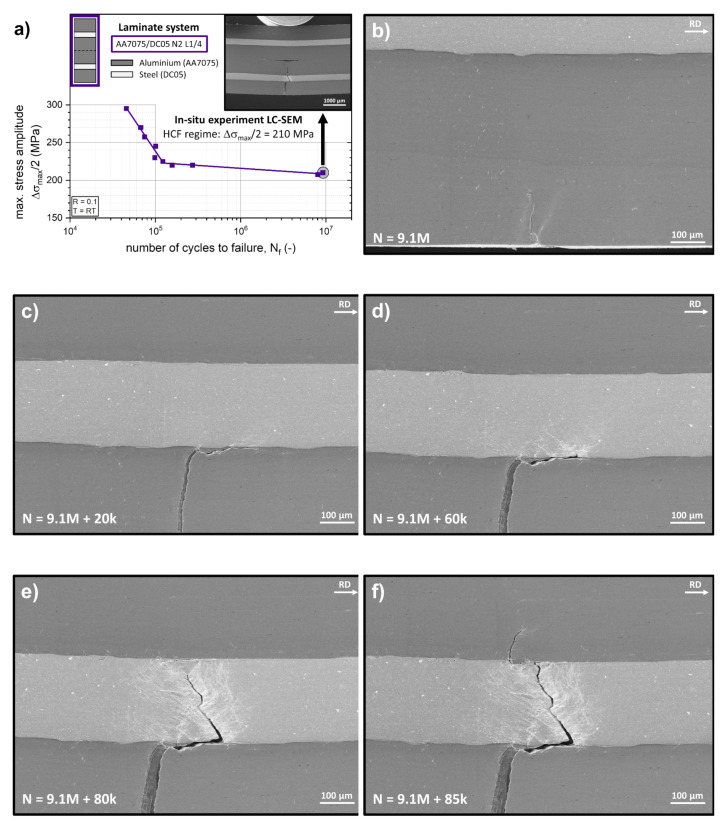
(**a**) Three-point bending fatigue experiment on a AA7075/DC05 laminated composite specimen fatigued in the HCF regime at a maximum stress amplitude of 210 MPa inside the large-chamber SEM. (**b**) Crack initiation occurring at 9.1 M cycles after external pre-fatigue on a vibrophore testing machine; (**c**–**f**) in-situ observation of the fatigue crack propagation perpendicular to the interfaces (crack arrester orientation).

A second in-situ fatigue experiment was conducted in the HCF regime at a maximum bending stress amplitude ∆*σ_max_*/2 of 210 MPa, as shown in Figure 10a. Prior to the in-situ experiment in the large chamber SEM, the specimen was pre-fatigued externally on a vibrophore testing machine at the same maximum bending stress amplitude of 210 MPa for 9.1 Mio cycles. The initiated crack after 9.1 Mio loading cycles using the method described above can be seen in Figure 10b. The crack was nucleated at the position of the maximum tensile stress in the outer AA7075 layer resulting from the cyclic three-point bending loading and propagates at a perpendicular orientation towards the interfaces. As in the LCF experiment, multiple small cracks originating at intermetallic particles can be seen on the front surface. No major contributions of these cracks on the propagation of the major crack were observed during the in-situ experiments. After 20 k additional loading cycles, the crack reaches the aluminium/steel interface (Figure 10c). As in the LCF regime experiment, a deflection of the crack path can be observed before the crack tip reaches the interface. Consequently, the crack approaches the interface at a high deflection angle and propagates along the interface for about 40 k loading cycles, as can be seen in Figure 10d.

At this stage, multiple events associated with local plasticity can be observed at different positions in the DC05 steel layer at the vicinity of the interface, indicated by secondary electrons contrast edge effects at roughened surfaces. Consequently, at one of these positions, a crack is nucleated into the adjacent steel layer. No formation of interface delamination cracks at the opposing steel/aluminium interface associated with stress redistribution effects was observed during this in-situ experiment. As can be seen in Figure 10e, after an additional 20 k cycles, the crack propagated through the steel layer at an angle, returning to the symmetry plane where the driving force on crack propagation is at a maximum due to the cyclic loading condition under three-point bending mode. After reaching the steel/aluminium layer, the crack deflects and propagates along the interface before penetrating the inner AA7075 layer (Figure 10f). This crack deflection at the steel/aluminium interface is much less pronounced when the length of crack that propagates along the interface (about 100 µm for the steel/aluminium interface vs. 250 µm for the aluminium/steel interface) is regarded. The less pronounced crack deflection is also visible by comparing the loading cycles for the crack propagation along the interfaces (below 5 k for the steel/aluminium interface vs. 60 k for the aluminium/steel interface). As for the loading cycle comparison, it must be stated again that the experiments were conducted under force control. The experiment was stopped once the crack reached the bonding plane of the final N2 ARB processing step. The crack network of this in-situ fatigued HCF specimen post-experiment, as can be seen in Figure 10a, resembles the crack network of the HCF fatigue-life specimen shown in Figure 8b, indicating an appropriate representation of HCF crack propagation using the described experimental procedure.

Using in-situ fatigue experiments, different toughening mechanisms associated with the interaction of the process zone ahead of the fatigue crack and the interfaces in laminated metallic composites with a combined gradient in hardness and elastic properties could be identified. The influence of these individual mechanisms was assessed in a semiquantitative manner. The findings can explain the enhanced fatigue crack propagation behavior of the laminated composites in the LCF regime associated with the prevalent toughening mechanisms.

### 3.4. Effects of the LMC Architecture Combined with Gradients of Hardness and Elastic Properties at Interfaces on Fatigue Life and Crack Propagation

Previous studies by the authors [15] revealed, that fatigue properties of LMCs can be specifically tailored by modifying the laminate architecture accordingly. In order to find optimized laminate architecture designs regarding fatigue life and crack propagation, laminates utilizing different combinations of material inhomogeneity effects in terms of gradients of hardness and elastic properties at interfaces were tested. The local mechanical properties in terms of hardness and Young’s modulus of the different AA2024, Ti-G1 and DC05 layers as well as the resulting gradients at interfaces can be found in Table 6.

Based on the respective hardness and elastic properties of the individual layers (Table 6), the highest gradients ∆*H* and ∆*E* in the laminated composite systems can be found at the AA2024/DC05 interfaces and the smallest gradients ∆*H* and ∆*E* at the AA2024/Ti-G1 interfaces, with the respective gradients of hardness and elastic properties of the Ti-G1/DC05 interfaces ranging in between.

**Figure 11:**
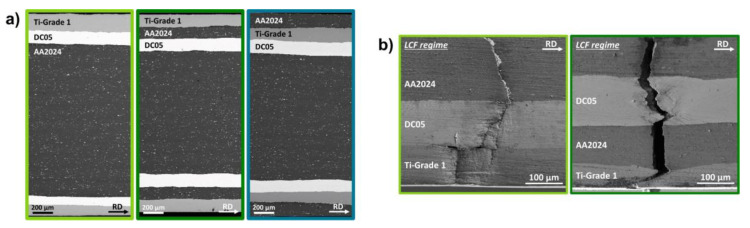
(**a**) SEM images of the tri-material laminated composite architectures Ti-G1/DC05/AA2024, Ti-G1/AA2024/DC05, and AA2024/Ti-G1/DC05, respectively; (**b**) Fatigue crack propagation paths in Ti-G1/DC05/AA2024 and Ti-G1/AA2024/DC05 LMCs fatigued in their respective LCF regimes.

Figure 11a shows the architectures of the different tri-material laminated composites investigated, containing thin layers of AA2024, Ti-G1 and DC05 near the respective surfaces (shell structure) as well as a AA2024 material core structure. The near surface AA2024, Ti-G1, and DC05 layers in the shell structure of the laminate have about the same thickness in average in all three laminate architectures, leading to the same nominal density for all LMCs.

**Figure 12:**
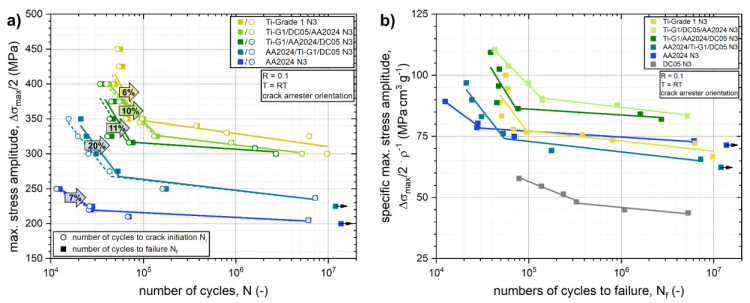
(**a**) Fatigue properties (*S-N* curves) of Al/Ti/Steel N3 LMC systems with different architectures (stacking sequences) and constituent monolithic Ti-G1 N3 and AA2024 N3 materials. The percentile of fatigue crack propagation on total fatigue life is indicated in the respective LCF regimes; (**b**) comparison of fatigue properties (*S-N* curves) of Al/Ti/Steel N3 LMC systems with different architectures and constituent monolithic Ti-G1 N3, AA2024 N3 and DC05 N3 materials in relation to their respective density *ρ*.

The fatigue life diagrams (*S-N* curves) of the Ti-G1/DC05/AA2024, Ti-G1/AA2024/DC05, and AA2024/Ti-G1/DC05 N3 laminate architectures as well as of the constituent AA2024 N3 and Ti-G1 N3 materials are plotted in Figure 12a. In all three laminated composite systems, the fatigue life in both the LCF as well as HCF regime can be improved significantly by the integration of thin titanium and steel layers compared to the monolithic AA2024 N3 material. A comparison of the *S-N* curves at the respective LCF to HCF region transition areas reveals a gradual enhancement in terms of endurable maximum stress amplitude and numbers of cycles to failure, indicating increasing levels of optimized laminate architecture designs regarding fatigue life properties.

The best resistance against crack initiation for the different LMC systems could be achieved in laminates where the titanium layers are positioned at the respective surfaces: Ti-G1/DC05/AA2024 and Ti-G1/AA2024/DC05. Comparing these two laminate architectures, the resistance against crack initiation is enhanced for the Ti-G1/DC05/AA2024 laminate architecture compared to the Ti-G1/AA2024/DC05 architecture. This can be explained again by the load transfer from the titanium surface layer into the adjacent DC05 steel layer due to the gradient in elastic modulus, thereby reducing the effective tensile stresses at the bottom surface titanium layer.

The best resistance against crack propagation was observed for the AA2024/Ti-G1/DC05 laminate architecture, where the crack propagation phase accounted for 20% of the numbers of cycle to failure in the LCF regime compared to 11% for the Ti-G1/AA2024/DC05 architecture, 10% for the Ti-G1/DC05/AA2024 architecture and about 6 to 7% for the monolithic Ti-G1 N3 and AA2024 N3 materials. This coincides with the findings presented in Section 3.1 and Section 3.3 and can be explained by a reduction of the local driving force at the crack tip, when the crack approaches the interfaces to the stronger and stiffer layers. For the AA2024/Ti-G1/DC05 architecture, this is the case for both the AA2024/Ti-G1 and the Ti-G1/DC05 interfaces (see Table 6), where the crack approaches the interface from the layer denoted first to the one denoted second, respectively. Figure 11b shows SEM images of the fatigue crack propagation paths through the laminated structure of the Ti-G1/DC05/AA2024 and Ti-G1/AA2024/DC05 LMCs fatigued in their respective LCF regimes.

In Figure 12b, the *S-N* curves are plotted in relation to the respective density ρ of the laminated composites and monolithic materials. The monolithic DC05 N3 steel exhibits by far the worst specific fatigue properties due to its high density. The specific fatigue life of the Ti-G1/DC05/AA2024 and Ti-G1/AA2024/DC05 LMC architectures are significantly improved compared to either of the constituent monolithic AA2024 N3, Ti-G1 N3, and DC05 N3 materials.

These findings clearly demonstrate that by an appropriate selection of the constituents, by adjusting their mechanical properties and by an intelligent design of the laminated metallic composite the fatigue life and cyclic crack propagation behavior of LMCs can be significantly improved.

## 4. Discussion

### 4.1. Effects of Toughening Mechanisms on Crack Propagation and Crack Growth in LMCs with a Hardness Gradient at Interfaces

The material inhomogeneity effect at the interfaces associated with plasticity was characterized as a hardness gradient at the interfaces by means of nanoindentation, since this method is well suited to determine local mechanical properties in thin layers of a laminated metal composite. The hardness gradients ∆*H* at the interfaces in the AA1050/AA5754 N2 LMC systems and the AA1050/AA5754 N3(Nxr) LMC system were found to be very similar at about 0.6 GPa, respectively.

As seen in Figure 4a, the resistance against crack propagation was improved for the Al/Al-LMC systems by reinforcing the softer matrix material with thin layers of a stronger material (16.7 vol.%) utilizing the hardness gradient effect at the interfaces. A better resistance against crack propagation was achieved for the AA1050/AA5754 L1/8 architecture. As can be derived from Figure 4b, the stronger AA5754 layer is positioned more towards the sample surface in the AA1050/AA5754 L1/8 architecture than in the AA1050/AA5754 L1/4 architecture. Figure 4a shows that the crack propagation phase amounts for 13% of total numbers of cycle to failure for the L1/8 architecture compared to 10% for the L1/4 architecture. Since the fatigue experiments were conducted under force control, the remote driving force for crack propagation depends on the remaining load bearing cross section of the specimen. If a crack approaches the interface towards the harder AA5754 layer, the process zone ahead of the crack tip is affected by the variation of local mechanical properties at the interface and thus decreases the crack tip driving force locally (crack tip shielding effect [35,40,41]). This effect is superimposed by the remote (far-field) driving force on the crack. This leads to the following explanation: For the L1/4 architecture, the contribution of the remote driving force is much bigger than for the L1/8 architecture when the crack approaches the vicinity of the interface, since the remaining load bearing cross section of the L1/4 architecture is much smaller compared to the L1/8 architecture by then. This means, the contribution of local effects on the overall crack tip driving force of a crack at vicinity of the interface is smaller for the L1/4 architecture, which leads to a smaller percentile of the crack propagation phase on the overall fatigue life of this laminate architecture.

A significant improvement of the damage tolerant fatigue properties of dissimilar Al/Al-LMCs with an alternating layer structure and an inherent hardness gradient ∆*H* at the interfaces of 0.6 GPa was observed conducting FCG experiments (Figure 5). As highlighted by post-experiment analysis of the surface crack networks using light microscopy (Figure 6) and analysis of the 3D-crack network using synchrotron X-ray computed microtomography (Figure 7), this can be attributed to the presence of two different toughening mechanisms observed at the interfaces: crack deflection and crack bifurcation.

Crack deflection was observed at the interfaces when the crack approached the interfaces from the softer to the harder layers. This toughening mechanism is associated with a deflection of the crack path at the vicinity of the interface and subsequent crack growth along the interface. Kümmel et al. [32] proposed a schematic evolution of the crack deflection mechanism based on their findings regarding crack propagation in fatigue life experiments on Al/Al-LMCs with dissimilar hardness. Similar correlations can be observed for the crack networks of the FCG experiments in Figure 6.

At low constant far-field stress intensity ranges (∆*K* = 7.5 MPa√m), no crack deflection mechanism could be observed. Similar to the HCF experiments by Kümmel et al., no deflection of the crack growth path at the vicinity of interfaces was observed, as the crack grows perpendicular across the interfaces. Although no deflection in the crack path was observed at ∆*K* = 7.5MPa√m, the crack growth rate in the AA1050/AA5754 LMC was determined to be about the same as for the monolithic AA5754 material and about 20% of the monolithic AA1050 material. This indicates that the periodic variation of the local mechanical properties and the associated variation of local crack driving forces in the laminated structures appears to have a retardation effect on the overall crack growth rate. With an increase in far-field ∆*K* level in Region II-1, the appearance of the crack deflection mechanism can be observed more frequently, indicating a dependence of this mechanism on the remotely applied far-field driving force for crack growth.

At higher constant far-field stress intensity ranges (∆*K* = 15 MPa√m and above), the crack deflection mechanism can be observed at every interface for the transition from the softer to the harder layers. The crack deflection mechanism can be explained as follows: When the crack approaches the interface from the softer material AA1050 to the harder material AA5754, the process zone ahead of the crack tip reaches the interface and is affected by the change of the mechanical properties in the adjacent layer. As this layer has a higher flow stress (higher hardness), the process zone ahead of the crack tip will be smaller at the same remotely applied far-field stress intensity range. This leads to a change of size and shape of the process zone at the vicinity of the interface. As the crack growth path depends on local conditions around the crack tip, the change of the characteristics of the process zone leads to a deflection of the crack path. Subsequently, crack growth in both directions along the interface was observed. The driving force for crack growth along the interface can be attributed to the formation of a complex stress state of compressional stress in the harder AA5754 layer and tensile stress in the softer AA1050 layer due to the geometrically necessary co-deformation behavior at the interface during fatigue loading [32]. This leads to stress redistribution mechanisms at the respective interfaces [27]. Similar findings for the crack deflection mechanism were previously observed experimentally by Sugimura et al. [31] and Pippan et al. [30] in Steel/Steel bi-metal and interlayer systems at the vicinity of interfaces for the transition of the crack from the softer to the stronger material.

For the soft/hard transition at interfaces in Al/Al-LMCs with dissimilar hardness, the following effects contribute to the overall crack growth behavior at the vicinity of these interfaces. These effects either reduce or accelerate the overall crack growth rate: (a) Crack tip shielding effect, reduction; (b) Deflection of the crack path and crack growth along interfaces, reduction; (c) Crack growth on multiple crack fronts in both directions along the interface, reduction.

Crack bifurcation was observed at the interfaces when the crack approaches the interface from the harder to the softer layers. This toughening mechanism leads to simultaneous crack growth at two separate crack fronts into the adjacent softer AA1050 layers (Figure 6).

At lower constant far-field stress intensity ranges (∆*K* = 7.5 MPa√m and 10 MPa√m), no crack bifurcation mechanisms could be observed, whereas at higher stress intensity ranges in Region II-1 (∆*K* = 12.5 MPa√m) and in Region II-2, this mechanism can be observed at multiple interfaces for the hard/soft transition in the respective crack networks.

For the time being, this mechanism is not fully understood. Two different considerations are suggested: The first consideration revolves around the anti-shielding effect [40,41]: When the crack approaches the interface from the harder to the softer material, the process zone ahead of the crack tip is affected by the change of the mechanical properties at the interface to the adjacent softer AA1050 layer. This leads to an enhancement of the process zone in size and a change in shape as can be seen in studies by Sistaninia et al. [39,78]. Consequently, the local crack tip driving force is enhanced significantly, as the crack reaches the interface to the softer material. This might lead to the crack bifurcation phenomenon and crack growth across multiple fronts inclined at 45–60° to the initial crack growth along regions of locally concentrated plastic deformation. Cyclic plastic deformation ahead of the crack tip is usually mainly concentrated in shear bands, inclined at an angle of 45–80° with regard to the direction of crack propagation [30]. The crack bifurcation mechanism was not observed experimentally at the respective interfaces in bi-metals [30,31]. Pippan et al. [30], however, observed a similar crack bifurcation phenomenon for crack growth into a softer α-Fe interlayer embedded in a ferritic steel matrix. This leads to the second consideration, where the crack bifurcation phenomenon might be linked to interaction of the process zone ahead of the crack tip with the second to next interface, that is the next soft/hard transition interface, when the crack approaches the interface of the hard/soft transition. This has two implications: (1) The crack bifurcation phenomenon would not be observable in bi-metals, as there is no second interface present and (2) the crack bifurcation phenomenon is only present in interlayer and laminated structures, when the process zone ahead of the crack tip is significantly larger for both materials than the respective layer thickness (that is at high far-field stress intensity levels).

In the literature, the delamination and bifurcation of cracks at interfaces are investigated numerically using cohesive zone models [79] or virtual crack closure techniques [80]. As shown by a recent numerical study using a cohesive zone model [81], a weaker interface strength and increased yield strength mismatch leads to an additional shielding effect of the crack tip driving force and can cause the crack to bifurcate at the interface. However, the numerically described bifurcation phenomenon deviates to some extent from the appearance of the bifurcation mechanisms observed in this study.

Although the mechanisms behind crack bifurcation are not fully understood, we can summarize the following effects that contribute to the overall crack growth behavior at the vicinity of the interfaces at a hard/soft transition in Al/Al-LMCs with dissimilar hardness: (a) The crack tip anti-shielding effect leads to an acceleration of the overall crack growth rate, while (b) crack bifurcation and simultaneous growth at multiple crack fronts results in its reduction.

### 4.2. Effects of Toughening Mechanisms on Crack Propagation in LMCs with a Combined Gradient in Hardness and Elastic Properties at Interfaces

The resistance against fatigue crack propagation in the LCF regime was improved for the Al/Steel-LMC system (Figure 8a) by reinforcing the softer and more compliant AA7075 matrix material with thin layers of a stronger and stiffer DC05 material (16.7 vol.%) utilizing gradient effect in hardness and elastic properties at the interfaces. In the HCF regime, the crack propagation phase plays a subsidiary role compared to the crack initiation phase. Consequently, the toughening mechanisms at the laminate interfaces do not contribute significantly to the overall fatigue life in the HCF regime.

**Figure 13:**
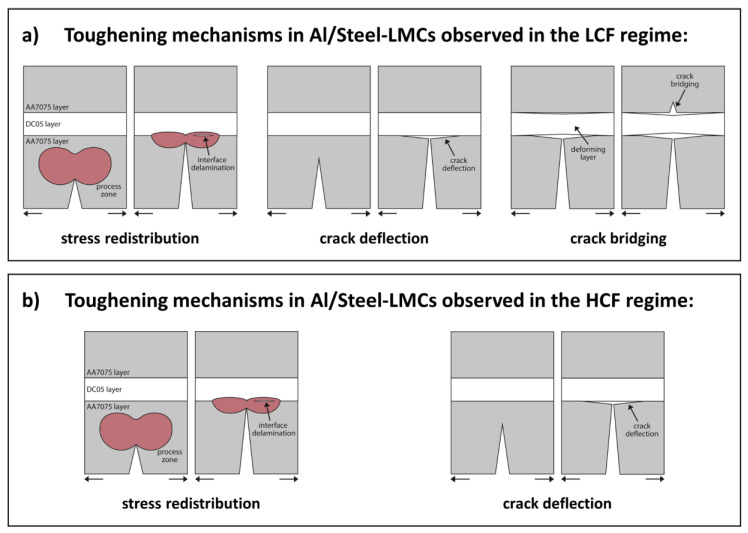
Toughening mechanisms in laminated aluminium/steel composites (AA7075/DC05) identified by means of in-situ fatigue experiments in three-point bending mode. Fatigue crack propagation perpendicular to the interfaces (crack arrester orientation). (**a**) LCF-regime: stress redistribution at interfaces leading to interface delamination, crack deflection at the interface towards the steel (DC05) layer, crack bridging of the deforming steel (DC05) layer; (**b**) HCF-regime: stress redistribution, crack deflection.

Figure 13 summarizes the prevalent toughening mechanisms observed by means of in-situ fatigue experiments in three-point bending mode in the LCF and HCF regime, respectively, motivated on the distinction of previously identified toughening mechanisms in LMCs by Lesuer et al. [27].

The crack deflection mechanism at the vicinity of the Al/Steel layer follows the same evolution as described in Section 4.1, however the crack shielding effect associated with the gradient in hardness (yield stress inhomogeneity effect) is enhanced by an additional shielding effect based on the elastic inhomogeneity effect [34] as the AA7075 layers are both softer and more compliant than the DC05 steel layers.

The stress redistribution mechanism is associated with the formation of a complex stress state at the Al/Steel interfaces due to the geometrically necessary co-deformation behavior as a response to the cyclic loading. This results in compressional stress in the stronger and stiffer steel layer and tensile stress in the softer and more compliant aluminium layers. This complex stress state leads to interface delamination ahead of the crack tip at high remote loadings (LCF regime, Figure 9d,e) and consequently promotes crack propagation along the interface of the deflected crack in both the LCF and HCF regime (Figure 9d and Figure 10d,f, respectively).

A crack bridging mechanism of the DC05 steel layer was identified at high remote loadings in the LCF regime (Figure 9e,f). On the macroscopic scale, this was observed in previous studies mainly for fatigue experiments on ductile/brittle laminate systems such as metal intermetal laminates (MILs), for example Nb/Nb_3_Al-MILs [23] and Ti/Ti_3_Al-MILs [24]. The crack bridging mechanism observed in this study is assumed to be closely linked to the stress redistribution mechanism at the Al/Steel interfaces, as interface delamination cracks are observed at the second interface (Steel/Al interface, Figure 9d,e). As explained above, inherent compressional stress in the steel layer and tensile stress in the aluminium layers are created due to the necessary co-deformation of layers with dissimilar (elastic) properties at the vicinity of the interfaces. This can explain the crack initiation into the inner AA7075 layer prior to the DC05 layer despite the remotely applied tensile stress level at the position of the steel layer being higher due to the loading in three-point bending mode.

Regarding the overall contribution of the individual mechanisms on the retardation of crack propagation at the vicinity of interfaces of materials with dissimilar hardness and elastic properties from the in-situ experiments, the following conclusions can be drawn: The biggest effect on retardation of crack propagation in the LCF regime can be attributed to shielding effects at the Al/Steel interface due to the yield stress inhomogeneity and elastic inhomogeneity effects. These effects lead to the appearance of the crack deflection and stress redistribution mechanisms. The effect of the crack bridging mechanism was not pronounced as much in terms of loading cycles. However, it must be stated that the tests were conducted under force control and the crack bridging of the steel layer occurred at a state where the load bearing cross section of the specimen was already significantly reduced leading to high remote driving forces for crack propagation.

### 4.3. Optimization of LMC Architectures for Light Weight Applications Subjected to Cyclic Loading Cases

The *S-N* curves in Figure 12 highlight the lightweight potential of laminated metallic composites by utilizing interface effects associated with gradients in hardness and elastic properties and integrating these effects into the laminate architecture design of LMCs. As can be seen in Figure 12a, the Ti-G1/DC05/AA2024 laminate architecture can endure almost the same number of cycles to failure in the LCF regime as the monolithic Ti-G1 material at a nominal composition of 12.5 vol-% Ti-G1, 12.5 vol-% DC05 and 75 vol.% AA2024. This is mainly attributed to the good resistance against crack initiation in the Ti-G1 surface layer and is supported additionally by load transfer into the adjacent stiff DC05 layer in cyclic three-point bending mode loading. As an additional effect, the resistance against crack propagation is enhanced from 6% for the monolithic Ti-G1 material to 10% of the total number of cycles for the Ti-G1/DC05/AA2024 laminate. This increases the interval of total fatigue life, where the presence of propagating cracks can be detected prior to failure. Due to the lower density of the LMC structures, the specific fatigue properties of the Ti-G1/DC05/AA2024 and Ti-G1/AA2024/DC05 laminate systems exceed those determined in monolithic Ti-G1 material (Figure 12b).

The resistance against crack propagation in the AA2024/Ti-G1/DC05 laminate architecture was increased to 20% of the total fatigue life in the LCF regime for tests conducted under force control in three-point bending mode by shielding effects attributed to yield stress inhomogeneity and elastic inhomogeneity effects at the vicinity of the Al/Ti as well as Ti/Steel interfaces. This emphasizes the potential of utilizing inhomogeneity effects at interfaces in laminated metal composites and the role of an intelligently designed LMC architectures in promoting damage tolerant properties.

## 5. Conclusions

The influence of gradients in hardness and Young’s modulus at interfaces in laminated metallic composites (LMCs) on fatigue crack propagation has been studied. The main conclusions are summarized in the following.

1. The resistance against crack propagation in the LCF regime is improved in Al/Al-LMC systems by reinforcing the softer AA1050 matrix with thin and harder AA5754 layers compared to monolithic AA1050 due to the hardness gradient at the interface.
2. The damage tolerant fatigue properties of dissimilar Al/Al-LMCs with an alternating layer structure are significantly enhanced in crack arrester orientation compared to both constituent monolithic AA1050 and AA5754 materials. Investigation of the surface and 3D-crack networks reveal the presence of two different toughening mechanisms at interfaces causing the reduction of crack growth in the LMCs. Crack deflection is observed when the crack approaches the interface from the softer towards the harder layers. Crack bifurcation happens in the opposite case. Both mechanisms appear to be more pronounced at higher remotely applied far-field stress intensity ranges. For the soft/hard transition at the interface in Al/Al-LMCs, a combination of the effects of crack tip shielding, deflection of the crack path and subsequent crack growth along the interfaces in both directions on multiple crack fronts contribute to the overall reduction of the local crack driving force at the vicinity of the interface. The local crack driving force at the vicinity of the hard/soft transition depends on the acceleration by anti-shielding effects and the superimposed reduction due to crack bifurcation and simultaneous crack growth along multiple crack fronts.
3. Resistance against crack propagation in the LCF regime is enhanced significantly in an Al/Steel-LMC system by reinforcing the softer and more compliant AA7075 matrix material with harder and stiffer DC05 steel layers, utilizing gradient effects in hardness and elastic properties at the interfaces. The prevalent toughening mechanisms observed in-situ are stress redistribution, crack deflection and crack bridging in the LCF regime and stress redistribution and crack deflection in the HCF regime. In the LCF regime, the enhanced crack propagation phase is attributed mainly to the crack tip shielding effects at the soft/hard and compliant/stiff transition at the Al/Steel interface, resulting in stress redistribution and crack deflection. The crack bridging of the steel layer appears to be promoted by interface delamination occurring at the inner Steel/Al interface due to stress redistribution and a complex stress state of compressional stress in the steel layer and tensile stress in the aluminium layers due to the geometrically necessary co-deformation of these layers at the interface upon cyclic loading.
4. The fatigue lives of Al/Ti/Steel tri-material laminated composites can be significantly improved compared to the monolithic AA2024 alloy by optimizing the laminate architecture, introducing thin Ti-G1 and DC05 layers. The specific fatigue properties of the LMC architectures containing Ti-G1 surface layers exceed those of the monolithic constituent materials significantly due to the lower density of the laminated composites. The effects of architecture optimization for laminated metal composites show promising potentials for LMCs in lightweight applications subjected to cyclic loading.

## Figures and Tables

**Table 1:** Chemical composition (wt.%) of the aluminium, steel, and titanium sheet metal, as measured by spark emission spectrometry.

Alloy	Chemical Composition (wt.%) ^1^											
	Al	Fe	Mg	Zn	Cu	Ti	Si	Cr	Mn	C	O	Others
AA1050	99.4	0.35	-	-	-	-	0.15	-	-	-	-	0.10
AA2024	93.4	0.10	1.42	-	4.37	-	0.07	-	0.46	-	-	0.18
AA5754	95.9	0.40	2.91	-	-	-	0.35	-	0.31	-	-	0.13
AA7075	89.4	0.12	2.72	5.77	1.53	-	0.07	0.20	-	-	-	0.19
DC05	-	99.7	-	-	-	0.07	-	-	0.10	0.01	-	0.12
Ti-Grade1	-	-	-	-	-	99.8	-	-	-	-	0.06	0.14

^1^ Elements with concentration below 0.05 wt.% listed as (-).

**Table 2:** Average hardness *H* of respective layers in the AA1050/AA5754 N2 LMC architectures as measured by nanoindentation measurements and resulting hardness gradient ∆*H* at LMC interfaces. Yield stress and ultimate tensile stress of the monolithic AA1050 N2 and AA5754 N2 materials as determined by uniaxial tensile tests.

Material	AA1050/AA5754 N2 LMC Systems		
	AA1050 Layers	AA5754 Layers	Gradient (∆*H*)
Hardness *H*/GPa	0.79 ± 0.07	1.37 ± 0.11	0.58 ± 0.13
**Material**	**Monolithic Materials**		
	**AA1050 N2 Mono**	**AA5754 N2 Mono**	**Difference**
Yield stress/MPa	152 ± 5	332 ± 5	180 ± 7
Ultimate tensile stress/MPa	172 ± 8	361 ± 2	189 ± 8

**Table 3:** Average hardness *H* of constituent monolithic AA1050 N2(N1r) and AA5754 N2(N1r) materials and respective layers in the AA1050/AA5754 N3(Nxr) LMC architecture as well as the resulting hardness gradient ∆*H* at the LMC interfaces as measured by nanoindentation measurements.

Material	AA1050 N2(N1r) Mono	AA5754 N2(N1r) Mono	AA1050/AA5754 N3(Nxr) LMC		
			AA1050 Layers	AA5754 Layers	Gradient (∆*H*)
Hardness *H*/GPa	0.71 ± 0.05	1.37 ± 0.10	0.73 ± 0.06	1.35 ± 0.12	0.62 ± 0.13

**Table 4:** Paris equation exponents *m* of Region II fatigue crack growth in monolithic AA1050 and AA5754 materials and a laminated composite AA1050/AA5754 in crack arrester orientation.

Material	AA1050 N2(N1r) Mono	AA5754 N2(N1r) Mono	AA1050/AA5754 N3(Nxr) LMC	
			Region II-1	Region II-2
Paris equation exponent *m*	3.37	4.51	0.89	5.72

**Table 5:** Average hardness *H* as measured by nanoindentation and Young’s modulus *E* of the respective AA7075 and DC05 layers in the AA7075/DC05 N2 L1/4 LMC architecture and resulting gradients ∆*H* and ∆*E* at interfaces. Yield stress and ultimate tensile stress of the monolithic AA7075 N2 and DC05 N2 materials as determined by uniaxial tensile tests.

Material	AA7075/DC05 N2 LMC System		
	AA7075 Layers	DC05 Layers	Gradients (∆*H*, ∆*E*)
Hardness *H*/GPa	1.55 ± 0.08	2.66 ± 0.12	1.11 ± 0.14
Young’s modulus *E*/GPa	70 ^1^	210 ^2^	140
**Material**	**Monolithic Materials**		
	**AA7075 N2 Mono**	**DC05 N2 Mono**	**Difference**
Yield stress/MPa	308 ± 6	575 ± 8	267 ± 10
Ultimate tensile stress/MPa	385 ± 4	620 ± 7	235 ± 8

^1^ data based on [74]; ^2^ data based on [75].

**Table 6:** Average hardness *H* as measured by nanoindentation and Young’s modulus *E* of the respective AA2024, DC05, and Ti-G1 layers in AA2024/Ti-G1/DC05 N3 LMC systems and resulting gradients ∆*H* and ∆*E* at interfaces. Yield stress and ultimate tensile stress of the monolithic AA2024 N3, Ti-G1 N3, and DC05 N3 materials, as determined by uniaxial tensile tests.

Material	AA2024/Ti-G1/DC05 N3 LMC Systems			
	AA2024 Layers	Ti-G1 Layers	DC05 Layers	Gradients (∆*H*, ∆*E*)
Hardness *H*/GPa	2.58 ± 0.05	2.78 ± 0.15	3.20 ± 0.13	Ti-Al: 0.20 ± 0.16
				Fe-Ti: 0.42 ± 0.20
				Fe-Al: 0.62 ± 0.14
Young’s modulus *E*/GPa	69 ^1^	103 ^2^	210 ^3^	Ti-Al: 64
				Fe-Ti: 107
				Fe-Al: 141
**Material**	**Monolithic Materials**			
	**AA2024 N3 Mono**	**Ti-G1 N3 Mono**	**DC05 N3 Mono**	**Difference**
Yield stress/MPa	639 ± 10	538 ± 4	554 ± 1	Al-Ti: 101 ± 11
				Fe-Ti: 16 ± 5
				Al-Fe: 85 ± 10
Ultimate tensile stress/MPa	654 ± 12	713 ± 2	704 ± 8	Ti-Al: 59 ± 12
				Ti-Fe: 9 ± 8
				Fe-Al: 50 ± 14

^1^ data based on [74]; ^2^ data based on [77]; ^3^ data based on [75].

## Data Availability

Not applicable.
